# Primary squamous cell carcinoma of the breast: A case report and review of the literature

**DOI:** 10.3389/fonc.2022.1033084

**Published:** 2023-01-06

**Authors:** Yaping Wu, Zhiying Chen, Wei Li, Fengguang Wang, Yongchun Zhang

**Affiliations:** Department of Radiation Oncology, The Affiliated Hospital of Qingdao University, Qingdao, China

**Keywords:** breast squamous cell carcinoma (BSqCC), breast metaplastic carcinoma, treatment, recurrence, case report

## Abstract

Primary squamous cell carcinoma (PSCC) of the breast, as a rare metaplastic breast cancer, currently has limited clinical data on its biological behavior, treatment and prognosis. At present, the optimal treatment of this tumor is still controversial. We reported a case of a 56-year-old woman with a mass on the right breast. She underwent a modified radical mastectomy and lymph node biopsy, which revealed that the tumor was a metaplastic squamous cell carcinoma with axillary lymph node metastasis, followed by traditional adjuvant chemotherapy and radiotherapy. The patient re-examined by PET/CT after two years in May 2017 and found a recurrence in the right chest wall, so resection of the recurring lesion was resected, then she was given postoperative adjuvant radiotherapy and chemotherapy. In August 2019, the patient re-examined by PET/CT, and there were pulmonary and mediastinal lymph node metastases. After 4 cycles of albumin paclitaxel plus cisplatin chemotherapy combined with nivolumab immunotherapy, the patient achieved complete response (CR), and then switched to nivolumab immune maintenance therapy. So far, no obvious metastasis has been seen. We believe that surgical treatment is necessary for PSCC of the breast;paclitaxel and cisplatin chemotherapy regimens and adjuvant radiotherapy are effective, but it may be resistant to radiotherapy; and immunotherapy may prolong the survival of patients with PSCC of the breast.

## Introduction

Primary squamous cell carcinoma (PSCC) of the breast is an extremely rare tumor, accounting for less than 0.1% of all invasive breast cancers (1, 2), and in 2012, the World Health Organization classified PSCC as metaplastic breast cancer. There are no standard treatment guidelines for PSCC of the breast, and most patients are treated with surgery, radiotherapy and chemotherapy, like breast ductal carcinoma. However, it is considered that breast PSCC is more malignant and aggressive than invasive ductal carcinoma of the breast (3). It is reported that the recurrence and metastasis of breast PSCC are common after treatment, and the prognosis is poor (4, 5). Here we present a case of a breast PSCC patient who had recurrences after radical mastectomy and adjuvant chemotherapy and radiotherapy. She had a long-term benefit from the use of immunotherapy combined with taxane and platinum-based chemotherapy.

## Case presentation

A 56-year-old postmenopausal woman went to the hospital because she found a lump on her right breast. She had no other clinical symptoms such as pain, skin change, nipple retraction, or nipple discharge. She denied family history of breast cancer and other tumors.

On physical examination, there was a 2 × 1.5 cm mass at 12 o’clock in the right breast, 0.5 cm away from the nipple. The lump was firm, movable and irregular. The rest of the physical examination showed no obvious abnormalities.

**Figure 1:**
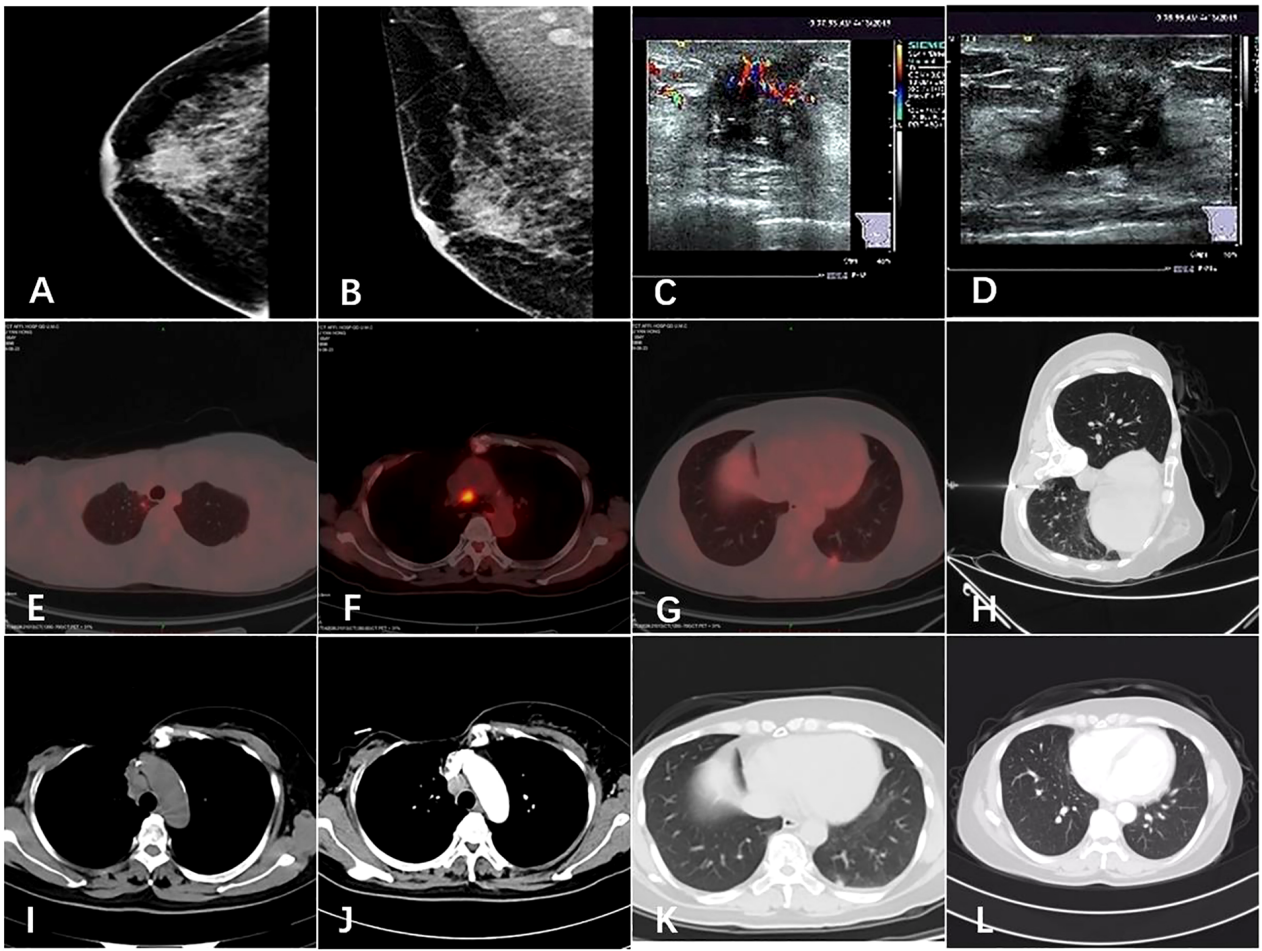
Imaging of the tumor. **(A, B)** Mammogram showed an irregular high-density shadow posterior to the nipple of the right breast and bilateral axillary lymph nodes were slightly enlarged. **(C, D)** Ultrasound showed a hypoechoic nodule on the right breast, 0.3 cm from the nipple at 12 o ‘clock, 1.7 × 1.4 cm in size. **(E, F, G)** The patient’s PET-CT results in 2019. Hypermetabolic lesions were found in the subpleura of the apical segment of the right upper lobe of the lung, the mediastinal anterior tracheal posterior vena cava lymph nodes and the subpleura of the basal segment of the left lower lobe. **(H)** Chest computed tomography guided needle biopsy of the left pulmonary nodule was performed. **(I, J, K, L)** Comparison of pulmonary nodules and mediastinal metastatic lymph nodes before and after the treatment.

The patient underwent diagnostic mammography and ultrasound to evaluate the right breast mass. The breast mammography showed (**Figures 1A, B**) abnormal density and calcification behind the right breast papilla, and bilateral axillary lymph nodes were slightly enlarged. Ultrasound of the breast showed (**Figures 1C, D**) insulting hypoechoic nodules. The patient underwent ultrasound-guided needle biopsy of the mass in the right breast, which revealed low-grade ductal carcinoma *in situ* with suspicious microinvasion.

**Figure 2:**
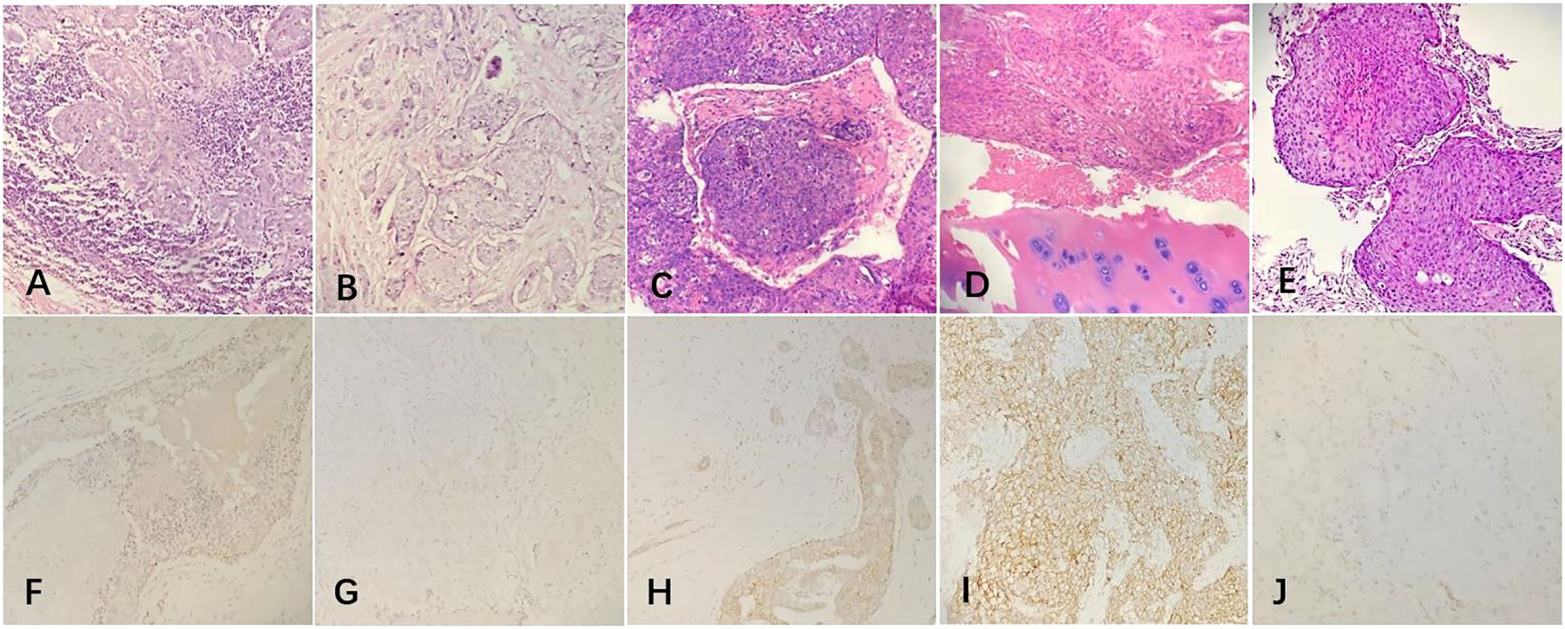
The postoperative pathological examination with hematoxylin and eosin (HE) staining and immunohistochemical (IHC) staining of the tumor. **(A, B)** Microscopically, the ductal cells in the mammary tumor area showed squamous degeneration and the lymph nodes were invaded by tumor cells (20x). **(C, D)** Histological findings with HE staining showed that the chest wall tumor was a moderately differentiated squamous cell carcinoma and the bone tissue was invaded by tumor cells(20x). **(E)** Histological findings with HE staining showed that the lung tumor was a metaplastic squamous cell carcinoma(20x). **(F–I)** IHC staining of the primary tumor in 2015. **(F)** ER negativity (20×). **(G)** PR negativity (20×). **(H)** HER2 receptor negativity (20×). **(I)** EGFR positive expression (20×). **(J)** IHC staining of the lung tumor showed that the PD-L1 positive.

The patient underwent a systemic examination to rule out distant metastases and other primary tumors. Then she underwent a modified radical resection of the right breast and a right axillary lymph node dissection on May 25, 2015. The final pathology showed (**Figures 2A, B**): (Right) Metaplastic carcinoma-squamous cell carcinoma (histological grade III, size 2.5*2.5*1.5cm) is seen in the upper and middle quadrant of the breast, interstitial vascular tumor thrombus (+);cancer metastasis can be seen in axillary lymph nodes (3/21); immunohistochemical showed (**Figures 2F–I**) estrogen receptor (ER) (-), progesterone receptor (PR) (-), human epidermal growth factor receptor 2 (HER2) (-), CK5/6 diffuse (+), CK14 diffuse (+), Ki67 positive rate 80%, P53 positive rate 90%,epidermal growth factor receptor(EGFR)(+), E-cadherin (-), P63 (+), D2-40 (+).After the operation, the patient received further adjuvant chemotherapy and radiation therapy. The specific chemotherapy regimen was a 4-cycle epirubicin combined with cyclophosphamide, followed by a 4-cycle docetaxel. After chemotherapy, radiotherapy was given. The scope of radiotherapy was the chest wall combined with the supraclavicular area; the dose was: 50Gy/25f, and the 95% isodose line was around the target area.

The patients received regular follow-up after the radiotherapy and chemotherapy. The PET/CT review on 2017-05-19 showed that: 1. There was a soft tissue mass on the right anterior chest wall (the inner side of the second anterior rib on the right), and the boundary between some layers and the right side of the sternum was not clear. Increased metabolism, standard uptake value (SUV)max was 4.0, considered chest wall metastasis and sternum invasion. The patient underwent a rib lesion resection under general anesthesia on May 26, 2017. Postoperative pathology showed (**Figures 2C, D**): (chest wall tumor) moderately differentiated squamous cell carcinoma. Combined with the medical history, the opinion was that the breast-derived metaplastic carcinoma (squamous cell carcinoma), nerve invasion (+), vascular invasion (+), estrogen receptor (ER) (-), progesterone receptor (PR) (-), human epidermal growth factor receptor 2 (HER2) (-), CK5/6 diffuse (+), Ki67 positive rate 60%, P40(+), P63 (+), the surrounding bone tissue was invaded, and the severed end was not involved. After the operation, 6 cycles of liposomal paclitaxel combined with carboplatin were given as chemotherapy. After chemotherapy, single-field electron irradiation was given. The irradiation range was ribs and sternum area. The dose was 54Gy/18f.

**Figure 3:**
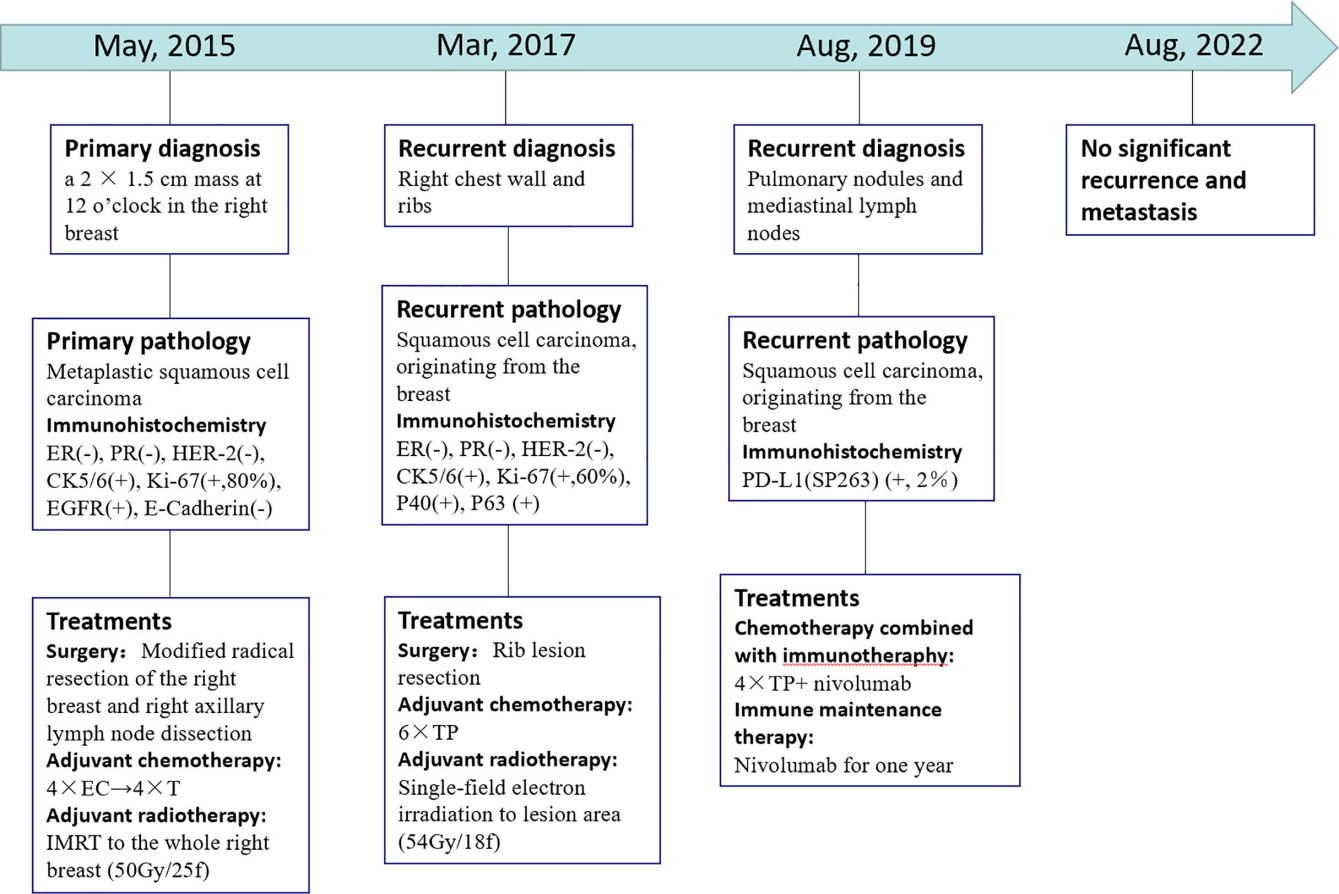
Timeline showcasing relevant data from the episode of care. (–): Negative expression; (+): Positive expression. 4 × EC→ 4 × T: four cycles chemotherapy of epirubicin and cyclophosphamide followed by four cycles of docetaxel. IMRT: intensity modulated radiotherapy. 6 × TP: Six cycles chemotherapy of liposomal paclitaxel and carboplatin. 4 x TP + nivolumab: four cycles chemotherapy of albumin paclitaxel plus cisplatin combined with nivolumab.

The patient’s PET/CT on August 23, 2019 showed (**Figures 1E–G**): 1. There were subpleural nodules in the posterior basal segment of the left lower lobe, and the metabolism was increased, the SUVmax was about 2.1; 2. There were small subpleural nodules in the apical segment of the right upper lobe, with increased metabolism, and the SUVmax was about 2.0; compared with the previous PET/CT examination, above lesions were new lesions, and metastasis was considered. 3. In the mediastinum, the anterior tracheal and retrocaval lymph nodes were slightly enlarged with increased metabolism, and the SUVmax was about 7.3, which was considered as lymph node metastasis. The patient underwent needle biopsy of the left pulmonary nodule (**Figure 1H**). The left lung nodule puncture pathology showed(**Figures 2E, J**) that the lung tissue had moderately differentiated squamous cell carcinoma infiltration, PD-L1(SP263) positive rate 2%,which was considered to be derived from breast cancer. We gave the patient an albumin paclitaxel plus cisplatin regimen for 4 cycles of chemotherapy combined with immunotherapy with nivolumab. After 4 cycles, the left and right lung nodules and mediastinal lymph nodes were basically invisible, and the curative effect was evaluated as CR (**Figures 1I–L**). Subsequently, the patient underwent nivolumab immune maintenance therapy for a year. So far, no obvious recurrence or metastasis has been found (**Figure 3**).

## Discussion

At present, there are few studies on PSCC of the breast. Its pathogenesis is still unclear, and there is a lack of standardized management of its molecular pathological characteristics, treatment and prognosis (6–8).

As a rare pathological type of breast cancer, some authors believe that it arises from benign breast disease (1, 9) and is seen in cystic epithelium, fibroadenoma, phyllodes tumor, papilloma, or chronic abscess. There are also some who suggest that phenotypic changes in breast cancer are the result of malignant transformation of breast cancer stem cells (histogenesis) or mutations in specific genes occurring early or late in carcinogenesis (dedifferentiation) (10). According to reports, radiotherapy can increase the occurrence of breast PSCC (11); in addition, implantation of prosthesis may be related to the occurrence of PSCC (12).

It is particularly important to make a correct diagnosis of PSCC of the breast. The current diagnosis of the disease believes that: the breast tumor cannot be derived from the skin of the breast or the skin of the nipple, or other attachments to the skin; the squamous cell carcinoma component in the tumor tissue accounts for more than 90%; excluding breast tumors derived from other primary tumors metastasized (13, 14). Metastatic breast cancer may be similar to primary breast cancer. Therefore, metastasis from other tumors should be excluded at the time of diagnosis. Testing for expression of CK7 and CK20 is considered to be the most beneficial. The great majority of primary breast cancers are CK7-positive and CK20-negative, whereas other metastases, such as colorectal cancers, are usually CK7-negative and CK20-positive. Aribas et al. similarly confirmed the diagnosis of breast metastases from colorectal cancer by immunohistochemistry (15).

A number of studies have found that the molecular expression of breast PSCC is significantly different from that of invasive ductal carcinoma. Most breast squamous cell carcinomas are ER-negative, PR-negative HER2-negative, CK5/6-positive, EGFR-positive, and highly proliferative (16).This “basal-like” phenotype can explain the poor prognosis of PSCC, even more aggressive than triple-negative breast cancer (17). The hormone receptors-negative and HER2-negative means that hormone therapy and HER-2 targeted therapy may not be effective in this tumor. In the future, the expression of high EGFR may bring new drugs to the treatment of PSCC of the breast (18).

It is reported that breast PSCC has a lower axillary lymph node metastasis rate (19). Previous studies have found that 10%-30% of breast PSCC cases have axillary lymph node metastasis, but blood spread and distant metastasis are more common (1, 13, 19). Also in other studies, compared with traditional breast cancer, the PSCC mass is larger, and part of it is a cystic mass of the breast (20, 21). The larger tumor size and less axillary lymph node metastasis suggest that PSCC may be suitable for modified radical mastectomy rather than breast-conserving surgery and sentinel lymph node resection is feasible for axillary lymph nodes.

For common breast cancer, if there are high-risk factors, adjuvant radiotherapy and chemotherapy are required after surgery. Our previous study showed that radiotherapy could reduce the local recurrence rate of breast PSCC, but the extent and dose of radiotherapy needed to be further confirmed (22). In a single institute series, in adjuvant radiotherapy for breast cancer, 6 out of 19 (32%) PSCC patients had local recurrences, and 4 of them had local recurrences in the irradiation field (3). In Mami Ogita’s multi-center study (23), 7 of 25 PSCC patients (28%) had local recurrence, of which 6 occurred at the irradiated site. It was found that a higher proportion of recurrences occurred in the irradiated area, concluded that breast PSCC may be radiation resistant. Therefore, increasing the radiation dose may be an option. Studies on chemotherapy regimens for PSCC lack large-scale clinical data. Some researchers have found that PSCC can benefit from platinum-based drug treatment, but is resistant to traditional breast cancer chemotherapy drugs such as anthracyclines and fluorouracils (24). Dejager et al. reported that cisplatin-based chemotherapy regiments normally used for squamous cell carcinoma in major organs other than the breast were also effective for squamous cell carcinoma of the breast (25). Rokutanda et al. and Murialdo et al. also reported the effectiveness of cisplatin-based chemotherapy in breast PSCC. After the patient relapsed after surgery, she chose to use paclitaxel combined with platinum-based chemotherapy, and both achieved good results (26, 27). Considering that breast PSCC may be the same as squamous cell carcinoma from other organs, we speculate that it is sensitive to taxans and platinum. Like the new drug albumin paclitaxel, whether it can further improve the efficacy remains to be studied on a large scale.

Currently, programmed cell death 1 (PDCD1, also known as PD-1)/programmed cell death 1 ligand 1 (PDCD1LG1, Pd-l1 (also known as PD-L1) immune checkpoint inhibitors for immunotherapy of breast cancer have gradually become a hot spot in clinical research, but there is a relative lack of research on the immunotherapy of primary squamous cell carcinoma. Using different antibodies and scoring systems, Muenst et al. found that PD-L1 was expressed in 152 of 650 breast cancer specimens (23.4%) (28). Doğukan et al. used immunohistochemistry to detect the expression of PD-L1 in 61 patients with triple negative breast cancer (TNBC), and the results showed that the positive expression rates of PD-L1 in tumor and tumor microenvironment were 37.7% and 47.5%, respectively (29). Sabatier et al. found that PD-L1 expression was associated with more aggressive subtypes (basal and erb-B2-rich), and in basal tumors, higher PD-L1 expression is associated with better PFS and OS and better response to chemotherapy (30). Studies have found that chemotherapy may enhance the release of tumor antigens, thereby enhancing the anti-cancer activity of immune checkpoint inhibitors. In particular, paclitaxel drugs can additionally activate the activity of toll-like receptors and promote the activity of dendritic cells (29). There have been several clinical trials of PD-1/PD-L1 inhibitors combined with chemotherapy in breast cancer, particularly in triple-negative breast cancer. In the Phase III clinical study of IMpassion130, patients with untreated metastatic triple-negative breast cancer were randomly assigned (in a 1:1 ratio) to receive atezolizumab plus albumin-bound paclitaxel or placebo plus albumin-bound paclitaxel treatment (31). The results showed that atezolizumab combined with albumin paclitaxel could further prolong the PFS and OS of triple-negative breast cancer, and the benefit was more obvious in the PD-L1 positive subgroup, and the adverse reactions of immunity and chemotherapy could be tolerated. After this patient developed lung and mediastinal lymph nodes metastases, we used nivolumab combined with albumin-bound paclitaxel and carboplatin. The lung and mediastinal lymph nodes metastases have achieved CR. Subsequently, immune maintenance therapy with nivolumab was administered for a year, and so far, the patient has not developed significant recurrence or metastasis. Most breast PSCC have a “basal-like” phenotype and the molecular phenotype is partially similar to triple-negative breast cancer, we speculate that breast PSCC may have a relatively high PD-L1 expression rate. Based on the efficacy of immunotherapy combined with chemotherapy in this patient and previous studies of immunotherapy in breast cancer, we believe that immunotherapy may also benefit patients with breast PSCC.

In conclusion, through the treatment of this patient, combined with previous studies, we believe that paclitaxel combined with platinum is a suitable chemotherapy regimen for breast PSCC. Adjuvant radiotherapy is beneficial, but it may be resistant, and increasing the dose of radiotherapy may further reduce the local recurrence rate. For advanced patients, chemotherapy combined with PD-1/PD-L1 inhibitors immunotherapy and immune maintenance therapy can further extend the patient’s survival, but it needs to be confirmed by large-scale clinical studies.

## Data availability statement

The original contributions presented in the study are included in the article/supplementary material. Further inquiries can be directed to the corresponding author.

## Ethics statement

The studies involving human participants were reviewed and approved by Medical Ethic committee of Affiliated Hospital of Qingdao University. The patients/participants provided their written informed consent to participate in this study. Written informed consent was obtained from the individual(s) for the publication of any potentially identifiable images or data included in this article.

## Author contributions

YW, ZC, WL, FW and YZ collected the patient’s data. YW and ZC finished the original manuscript and YW revised it. WL and FW provided the figures. YZ provided final approval for the version to be published. All authors contributed to the article and approved the submitted version.
